# Quantitative modelling for dengue and *Aedes* mosquitoes in Africa: A systematic review of current approaches and future directions for Early Warning System development

**DOI:** 10.1371/journal.pntd.0012679

**Published:** 2024-11-26

**Authors:** Lembris Laanyuni Njotto, Wilfred Senyoni, Ottmar Cronie, Michael Alifrangis, Anna-Sofie Stensgaard

**Affiliations:** 1 College of Information and Communication Technologies, University of Dar Es Salaam, (CoICT—UDSM), Dar Es Salaam, Tanzania; 2 Department of Mathematics and ICT, College of Business Education, Dar Es Salaam, Tanzania; 3 Department of Mathematical Sciences, Chalmers University of Technology & University of Gothenburg, Gothenburg, Sweden; 4 Department of Immunology and Microbiology, Centre for translational Medicine and Parasitology, University of Copenhagen, Copenhagen, Denmark; 5 Department of Infectious Diseases, Copenhagen University Hospital, Copenhagen, Denmark; 6 Section for Parasitology and Aquatic Pathobiology, Department for Veterinary and Animal Sciences, University of Copenhagen, Copenhagen, Denmark; Kenya Agricultural and Livestock Research Organization, KENYA

## Abstract

The rapid spread and growing number of dengue cases worldwide, alongside the absence of comprehensive vaccines and medications, highlights the critical need for robust tools to monitor, prevent, and control the disease. This review aims to provide an updated overview of important covariates and quantitative modelling techniques used to predict or forecast dengue and/or its vector *Aedes* mosquitoes in Africa. A systematic search was conducted across multiple databases, including PubMed, EMBASE, EBSCOhost, and Scopus, restricted to studies conducted in Africa and published in English. Data management and extraction process followed the ‘Preferred Reporting Items for Systematic Reviews and Meta-Analyses’ (PRISMA) framework. The review identified 30 studies, with the majority (two-thirds) focused on models for predicting *Aedes* mosquito populations dynamics as a proxy for dengue risk. The remainder of the studies utilized human dengue cases, incidence or prevalence data as an outcome. Input data for mosquito and dengue risk models were mainly obtained from entomological studies and cross-sectional surveys, respectively. More than half of the studies (56.7%) incorporated climatic factors, such as rainfall, humidity, and temperature, alongside environmental, demographic, socio-economic, and larval/pupal abundance factors as covariates in their models. Regarding quantitative modelling techniques, traditional statistical regression methods like logistic and linear regression were preferred (60.0%), followed by machine learning models (16.7%) and mixed effects models (13.3%). Notably, only 36.7% of the models disclosed variable selection techniques, and a mere 20.0% conducted model validation, highlighting a significant gap in reporting methodology and assessing model performance. Overall, this review provides a comprehensive overview of potential covariates and methodological approaches currently applied in the African context for modelling dengue and/or its vector, *Aedes* mosquito. It also underscores the gaps and challenges posed by limited surveillance data availability, which hinder the development of predictive models to be used as early warning systems in Africa.

## Author summary

Infections from dengue and other arboviral mosquito-borne diseases transmitted by *Aedes* mosquitoes are on the rise globally, with Africa being no exception. Their advances are driven by anthropogenic factors, such as rapid urbanisation, globalisation, and climate change. Yet, knowledge of dengue epidemiology and burden on the African continent, and how to enhance preparedness is scarce. Navigating the complexities of predicting the spread/outbreaks of the dengue or the presence/abundance of *Aedes* vector mosquitoes, is challenging due to the complex interactions between multiple factors involved in the transmission. Despite these challenges, significant progress has been made in developing various quantitative methods to predict spread and outbreaks in different regions in the world. Here, we conducted a systematic review to shed light on existing quantitative modelling approaches for dengue and/or its vector *Aedes* mosquitoes in Africa, focusing on methodology, data sources, covariates used, model performance and validation. Our study revealed several shortcomings in current modelling practices in Africa and emphasized the need for real-time primary predictor data and more comprehensive reporting of model development techniques and validation processes. This review offers an evidence-based framework for improving future modelling practices, to develop more accurate and robust dengue prediction models, tailored for African contexts.

## Introduction

Dengue is currently the fastest-spreading arboviral mosquito-borne disease globally, with a high morbidity in children and adults in many tropical and sub-tropical countries. It stems from the dengue virus (DENV), a flavivirus prevalent in 128 countries, infecting an estimated 390 million individuals annually [1–4]. Over the past two decades, the number of globally reported human DENV cases has increased by more than a factor of 10 [5]. Urbanization, rapid population growth, increase in international travel and trade, deficiency in vector control strategies, inadequate public health infrastructure and climate change have been identified as important contributors to this resurgence of dengue [5–7].

On the African continent, more than 20 countries have reported outbreaks of dengue since the 1960s, and the prevalence of dengue appears to have dramatically increased over the past few decades, despite neither being systematically investigated nor generally considered as a possible cause of fever by clinicians [8,9]. Africa’s growing populations, rapid and unplanned urbanization, increasing global trade and movement of goods and people, makes it a rich spawning ground for the spread of arboviruses such as DENV across the continent [10]. Still, the epidemiology and burden of dengue are largely uncharacterized. Historically overshadowed by malaria, preparedness and early warning systems in Africa for arboviral diseases such as dengue that are transmitted by a different genus of mosquito, thus remains comparatively underdeveloped [11].

Preparedness, including the development of prediction models for dengue outbreaks, is closely linked to the biology and ecology of the vector mosquitoes of the genus *Aedes*. *Aedes aegypti* is recognized as the primary vector for the DENV in Africa, while *Ae*. *albopictus* is considered more invasive due to its ability to thrive in a wider range of environments, including both urban and rural areas [12–14]. *Ae*. *albopictus* can breed in a variety of natural and artificial containers, making it highly adaptable and capable of spreading rapidly [15,16]. In contrast, *Ae*. *aegypti* is typically more associated with urban environments [15,17], where it breeds in containers with clean water. The adaptability of *Ae*. *albopictus* to diverse habitats contributes to its invasive potential, allowing it to establish populations in regions where *Ae*. *aegypti* might be less prevalent [18].

The development of *Aedes* from egg to larval and adult stages is heavily influenced by climatic variables such as temperature, precipitation, and relative humidity [19–21]. Increased temperature (up to a certain limit) can quicken mosquito development, shorten the time between blood meals, and affects the virus structure, resulting in increased transmission [22,23]. *Aedes* mosquitoes thrive in temperatures ranging from 15°C to 35°C [12,24]. At the same time, adult mosquito activity and survival are similarly influenced by humidity since they are more active and live longer in humid environments [25]. Rainfall also influences dengue transmission to a very high degree because it offers breeding grounds for mosquitos to lay eggs, increases mosquito population, and regulates temperature and humidity, both of which are essential for mosquito survival [22,26]. In addition to the direct influence of climatic variables on the development and survival of *Aedes* mosquitoes, the concept of lag effects plays a crucial role in understanding mosquito population dynamics and disease transmission. Lag effects refer to the delayed response of mosquito populations and disease incidence to changes in climatic conditions. For instance, a period of increased rainfall might not immediately result in an increase in mosquito populations or dengue cases but could lead to a significant increase weeks later as the eggs laid during the wet period hatch and develop into adults. Similarly, changes in temperature might affect mosquito development rates and viral replication with a delay, influencing transmission dynamics after a certain period. Studies have shown that temperature and precipitation lags of one to several weeks can significantly impact mosquito abundance and the timing of dengue outbreaks [27–29]. These lags are important to consider when modelling disease transmission, as they help in predicting outbreaks more accurately by accounting for the time it takes for climatic changes to translate into increased mosquito activity and disease risk.

In the fight against dengue transmission, in Africa as well as globally, it is crucial to implement effective prevention measures, particularly in the absence of comprehensive vaccines and medications. Among these strategies, vector control targeting the mosquito has demonstrated a considerable success in mitigating dengue outbreaks [30]. However, the traditional practices often rely on reactive responses, waiting to observe an increase in the number of cases before identifying potential outbreaks. This reactive approach poses limitations, as it may lead to delayed responses, allowing outbreaks to escalate before interventions are applied. To address this challenge, there is a growing emphasis on developing forecasting models to serve as early warning systems (EWS) for dengue outbreaks. Such models should be capable of predicting disease outbreaks before they occur, identifying high-risk areas or populations prone to infection, and integrating both spatial and temporal dimensions. This model should incorporate real-time data, such as climatic conditions, vector population dynamics, human mobility patterns, and historical disease data, to provide timely and accurate predictions. By leveraging these predictive insights, proactive measures can be implemented swiftly to prevent outbreaks from worsening, thereby safeguarding public health and reducing the risk of disease transmission.

In the realm of quantitative prediction techniques for dengue outbreaks, a wide range of modelling approaches has been explored, including statistical, mathematical, and machine learning models [31–35]. These varieties of approaches in the “modelling toolbox” each has its own strengths and weaknesses, with a distinct purpose. The challenge arises from the involvement of multiple factors, including the DENV themselves, the vector mosquitoes, and the populations susceptible to infection [36,37], reflected in a wide divergence among models in terms of their setups and goals. To be effective in predicting outbreaks, a model needs to be adaptable and capable of connecting susceptible population with weather patterns across different geographical regions [38], while also capturing the temporal aspect by predicting risks or outbreaks in close to real-time and identifying higher-risk populations [39]. Selecting the appropriate components, or covariates, for these models poses another challenge. Some models prioritize climate variables [40,41], while others consider mosquito characteristics or human population demographics [42–44]. The debate over “the best model” is ongoing, especially in the African context, where a lack of capacity for arbovirus outbreak preparedness, surveillance, and control has been highlighted [11]. Our focus on Africa is driven by a gap in knowledge to understand the true scale and drivers of dengue in Africa, as well as the region’s unique climatic and socio-economic conditions, significantly influencing dengue transmission dynamics. Africa faces specific environmental and public health challenges that differ from other parts of the world, necessitating a focused analysis. Expanding the study globally could dilute the attention on these critical factors. Additionally, the global perspective has already been considered in other studies [33,45,46], making our regional focus particularly relevant for addressing gaps in understanding dengue in the African context.

Here, using a broader definition of “dengue modelling”, we conducted a systematic review of all existing quantitative models applied to either dengue (i.e. human cases), as well as models aimed at predicting or explaining *Aedes* vector distribution or abundance (as indicators of potential dengue risk/outbreaks) in an African context. The aim was to identify and assess a) the methods, sources of data, and key findings of the published modelling studies, and b) specific influential environmental factors and other factors associated with dengue risk/outbreaks and/or *Aedes* mosquitoes on the continent. Finally, based on the identified modelling approaches applied in the African context, we evaluate potential obstacles and possible ways forward towards the development and implementation of EWS for dengue in the African realm.

## Methods

The systematic review’s objective, search strategy, and inclusion and exclusion criteria were crafted following the ‘Preferred Reporting Items for Systematic Reviews and Meta-Analyses’ (PRISMA) framework, to ensure that the methodologies utilized for the systematic review were clear, transparent, and consistent [47]. The PRISMA framework provides guidelines for conducting systematic reviews and meta-analyses, thereby enhancing the quality and transparency of reporting in such studies. All figures and maps were generated using the R software (version 4.3.3) [48].

### Databases and search strategy

The literature search encompassed articles published until June 2023, ensuring a comprehensive and up-to-date exploration of relevant studies. Four electronic databases, namely PubMed, EMBASE, EBSCOhost, and SCOPUS, were employed to search and retrieve all published articles using the search terms outlined in **S1 Table.** To enhance the sensitivity and specificity of the initial search across various databases, combinations of keywords were employed. Additionally, thematic keywords were refined using Boolean operators and truncations before being applied to the selected electronic databases. Grey literature, such as commentaries, reports, and expert reviews that did not present original research, were consulted for additional information. The resulting relevant studies were then imported into an Excel database for further analysis.

### Inclusion and exclusion criteria

The articles selected for this review were chosen based on specific inclusion and exclusion criteria. Firstly, we included only available peer-reviewed articles that presented a quantitative model (predictive or explanatory) of dengue infections, including lab-confirmed infections, IgG/IgM seropositivity for dengue, dengue or dengue haemorrhagic, and overall dengue prevalence, hereafter referred to as “dengue models” for the remainder of the paper. We also considered articles which used models to explain or predict the population dynamics of dengue vectors (e.g., *Ae*. *aegypti* or *Ae*. *albopictus*), as a proxy for dengue risk, hereafter referred to as “mosquito models” for the remainder of the paper. A quantitative technique, in this context, refers to a systematic and measurable approach that involves the use of numerical data and the application of statistical methods, mathematical algorithms, or computational tools to analyse and interpret these data. Secondly, our search was restricted to publications conducted in any African country and presented in the English language. There were no exclusions based on a study’s design or publication year.

### Data extraction

The initial screening process involved an assessment of study titles and abstracts to determine their relevance. Studies that aligned with the research objectives were then subjected to further evaluation to ascertain their eligibility for full-text review. Next, during the full-text review, a more stringent set of inclusion and exclusion criteria were employed to select studies for data extraction. This process involved extracting detailed information covering several key aspects:

- study identification (study titles, author names, publication year, and study location);
- quantitative model characteristics (type of model used and their data sources including either human dengue cases or the population dynamics of *Aedes* vectors mosquito, the covariates included in these models and their respective data sources);
- model assessment (variable selection approaches, model validation and performance metrics).

Furthermore, the reference lists of the identified studies were examined to identify any supplementary relevant papers. The searches were conducted and double-checked until consistent results were achieved. Two authors (LLN and ASS) reviewed all hits to determine their relevance. Subsequently, one data extractor (LLN) evaluated the abstracts and full texts of the selected references for potential eligibility by applying all inclusion and exclusion criteria. All relevant studies were then imported into Microsoft Excel 365 (Version 2303), where essential details from each chosen study were extracted, as listed above. The extracted data from these studies were then summarized, and the methodological characteristics of the models were organized into a table.

## Results

### Literature retrieval and characteristics of the included studies

**Fig 1:**
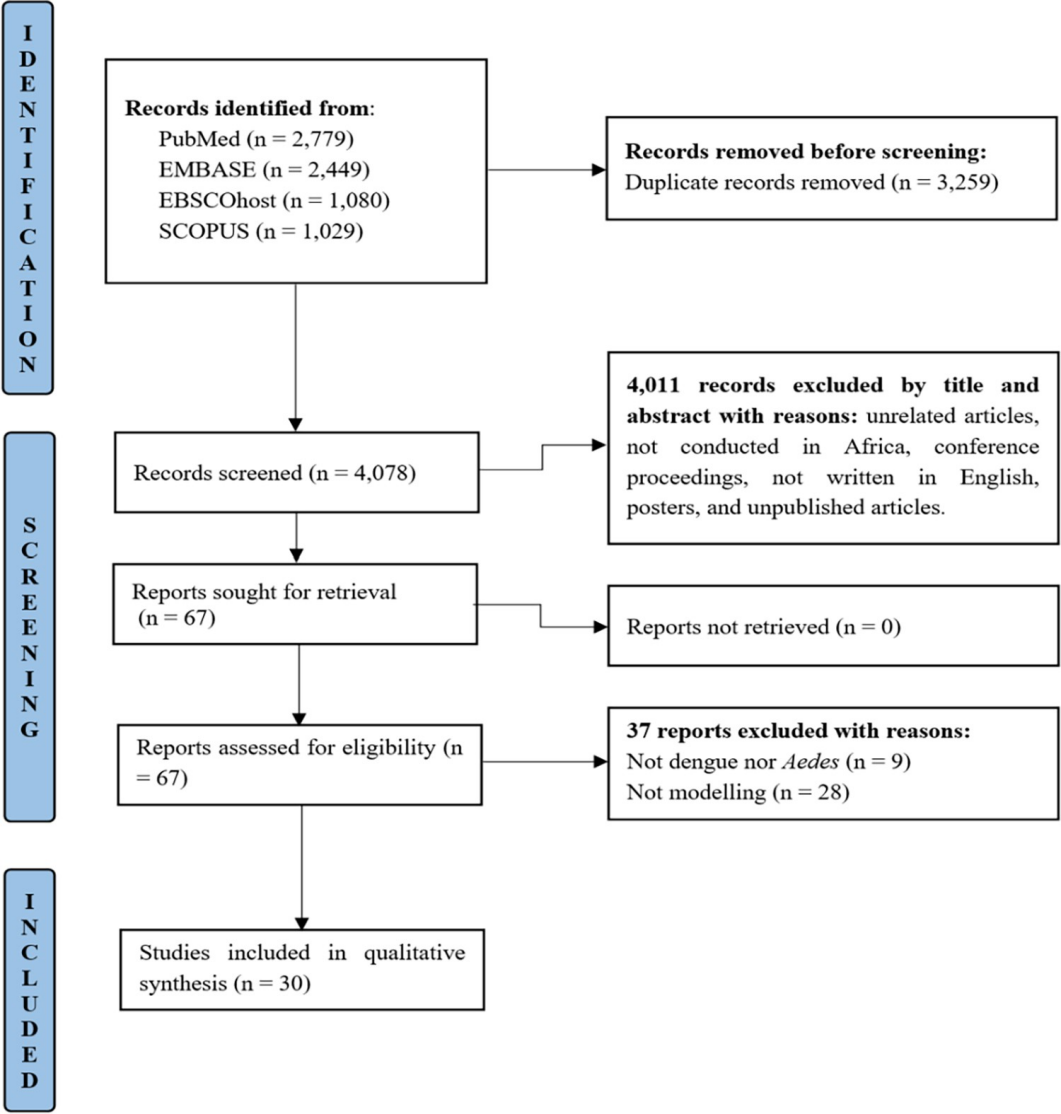
PRISMA Flowchart depicting the number of records identified, included and excluded, as well as the reasons for exclusion.

Based on keyword searches, a total of 7,337 records were identified during literature retrieval from databases. After removing 3,259 duplicates, 4,078 records had their titles and abstracts screened. Subsequently, 67 articles underwent full-text review, and after evaluating them for eligibility based on inclusion and exclusion criteria, 30 articles were retained. The full article screening and selection process is depicted in the PRISMA flowchart (**Fig 1**).

A summary of the identified covariates used in the studies are provided in **Table 1**. Details of the studies are provided in **S2 Table**, encompassing the key identifiers and aspects considered in the identified studies. It includes whether the primary goal was to model or predict human dengue cases/incidence (referred to as “dengue models”) or population dynamics of *Aedes* vector mosquitoes (i.e., abundance or presence) as outbreak indicators (referred to as “mosquito models”). Additionally, it details the sources of input data, the geographical coverage of the studies, and the types and numbers of covariates incorporated in the models, which include environmental, climatic, larval/pupal abundance, demographic, and socioeconomic factors, along with their sources. The table include details about the types of quantitative techniques applied in the models and the metrics employed for both variable selection and model validation.

**Table 1:** Types of covariates included in the identified modelling studies, and their distributions. The percentages given are calculated as the total number of studies utilizing the covariate in question out of the total number of identified modelling studies in the systematic review.

Covariates	Number of Studies		Total percentage (n/30)
	Dengue models	Mosquito models	
**Climatic factors**			
Temperature	2	5	23.3%
Mean Temperature		5	16.7%
Minimum Temperature	2	1	10.0%
Maximum Temperature	2	1	10.0%
Rainfall/precipitation (total)	2	4	20.0%
Bioclimatic variables		5	16.7%
Relative Humidity	2	3	16.7%
Seasonal (Dry, Rainy)		4	13.3%
Sun exposure		3	10.0%
Wind speed	1		3.3%
**Environmental factors**			
Mosquitoes collection location (indoors/outdoors)		6	20.0%
Vegetation		6	20.0%
Habitant type/count		4	13.3%
Elevation/altitude		2	6.7%
Location of breeding site (urban/peri-ban/rural)		2	6.7%
Distance to water bodies		1	3.3%
**Demographic factors**			
Age	5	1	20.0%
Gender	5	1	20.0%
Travel outside the country	2		6.7%
Population density		2	6.7%
Vaccination		1	3.3%
**Socio-economic factors**			
House type or construction materials	5	1	20.0%
Use of mosquito preventive measures	4	1	16.7%
Household density/status	3	1	13.3%
Education level/status	3		10.0%
Occupation and employment status	3		10.0%
Access to clean water		2	6.7%
**Larval/pupal abundance factors**			
House Index (HI)		4	13.3%
Container Index (CI)		4	13.3%
Breteau Index (BI)		4	13.3%
Pupae Index (PI)		3	10.0%
Pupae per Person Index (PPI)		1	3.3%

### Distribution of dengue and *Aedes* modelling studies in Africa

**Fig 2:**
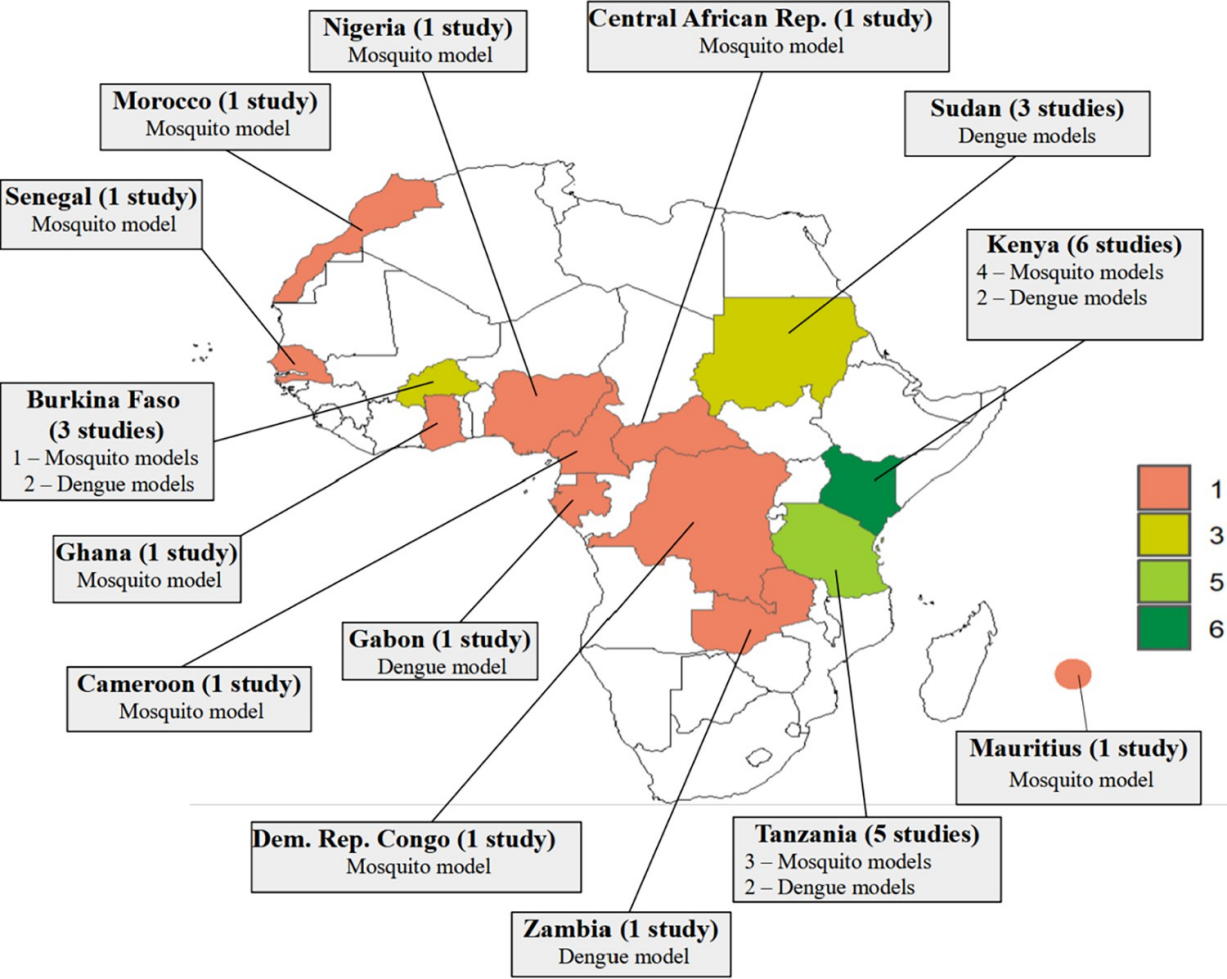
Map of Africa displaying the geographical distribution and scope of the studies and categories of quantitative models (dengue models and mosquito models) as revealed by our systematic review. The Africa shapefile was obtained and mapped in R using the naturalearth, rnaturalearthdata, and ggplot2 packages.

The selected studies demonstrate a significant geographical diversity across multiple African countries (**Fig 2**), with a notable concentration of studies originating from East Africa. The country with most studies was Kenya, with 6 studies conducted (out of which 4 were mosquito models [49–52] and 2 were dengue models [53,54], while the Republic of Tanzania contributed 5 studies, including 3 on mosquito models [55–57] and 2 on dengue models [58,59]. Finally, three studies were conducted globally, accessing the distribution or global risk for major disease transmitted by *Ae*. *aegypti* and *Ae*. *albopictus* across multiple countries and including over 50 African countries and territories (60–62) (not depicted on the map in **Fig 2**).

### Type of data used as model outcome

Most of the identified models employed in Africa, about two-thirds (19 studies out of 30), focused on modelling the presence or abundance of *Aedes* mosquitoes (“mosquito models”) as an indicator or proxy for assessing the risk of dengue occurrence or outbreak, while only one-third (11 studies out of 30) of the models attempted to directly model the actual risk of dengue by assessing the human dengue cases/incidence (“dengue models”). The outcomes or responses in these dengue models included laboratory-confirmed cases (included by 5 studies), dengue/dengue IgM prevalence/seropositivity (included by 3 studies), and dengue infections (considered by 3 studies). For mosquito models, the abundance of mature/immature *Ae*. *aegypti or Ae*. *albopictus* was considered by 10 studies, the presence of mature/immature *Ae*. *aegypti or Ae*. *albopictus* was utilized by 5 studies, mosquitoes infected with the dengue virus were utilized by 2 studies, and viral transmission risk and ovitrap positivity were each utilized by one study. None of the studies focusing on dengue models applied a predictive modelling approach, while six studies [57,60–64] related to vector mosquito modelling had a predictive purpose. Most of the studies were primarily conducted at regional and district levels (36.7%), with 20.0% conducted in cities. Additionally, 10.0% of all the identified studies had a multicounty scope, while the remaining studies were conducted at the state, provincial, village, zone, port, and national levels.

In the analysed studies, the sources of dengue and entomological data used to model dengue risk or outbreak indicators varied considerably. These sources included information on human dengue cases and *Aedes* mosquito abundance. Most of the studies (66.7%) relied on entomological surveys (43.3%) and cross-sectional surveys (23.3%) as their primary sources of dengue infection and mosquito data, respectively. Approximately 20% of the studies used government records for dengue infection data, sourcing information from Ministries of Health, hospital surveillance, and health statistical yearbooks to obtain the number of weekly or monthly laboratory-confirmed dengue cases. Conversely, 6.7% (2/30) of the studies on mosquito data used secondary data from previously published studies, while another 6.7% (2/30) utilized global data from the Global Biodiversity Information Facility (GBIF) and from World Health Organisation (WHO) weekly epidemiological reports.

### Model covariates

The reviewed studies incorporated a diverse set of covariates into their models, grouped into five distinct categories: climatic, environmental, demographic, socioeconomic, and larval/pupal abundance factors. The number of covariates utilized was counted based on their use in either mosquito or dengue models, as shown in **Table 1.** Most studies incorporated at least one of these categories into their models, but three studies [60,65,66] did not include any of these covariates at all. Most of the dengue models incorporated climatic, demographic, and socioeconomic factors, but did not include any covariates from environmental and larval/pupal abundance factors. In contrast, most mosquito models incorporated at least one covariate from all five categories. The majority of these covariates (43.3%) were obtained through entomological and cross-sectional surveys, with 10.0% obtained from local meteorological weather stations and national databases, and 30.0% from global databases such as the Global Population Database, the National Oceanic and Atmospheric Administration (NOAA), NASA’s Earth Observing System (EOS), and WorldClim.

### Climatic and environmental factors

Climatic factors were the most included group of variables in the models, with temperature being the most frequently used, followed by rainfall. About 23.3% of the studies (involving 2 dengue models and 5 mosquito models) incorporated temperature, while 20% of them (involving 2 dengue models and 4 mosquito models) accounted for rainfall. Among temperature metrics, mean temperature was the most utilized, appearing in 16.7% of the studies. All studies reviewed considered total or cumulative rainfall in their models. The primary sources of data for temperature (4 studies) and rainfall (3 studies) were local meteorological stations. There was a limited use of satellite data across the reviewed studies, and these included the use of satellite databases, such as NASA’s Earth Observing System (EOS) (67), WorldClim [62], and the National Oceanic and Atmospheric Administration (NOAA) [50,68].

### Climatic and environmental factors for dengue models

A study conducted in Burkina Faso revealed that relative humidity, maximum and minimum temperatures, and wind speed had a significant non-linear effect on dengue cases [67]. They found that the optimal temperature for increasing dengue cases was between 27°C to 32°C for the maximum temperature and between 18°C to 20°C for the minimum temperature. The study also indicated that the estimated number of dengue cases increased in two distinct ranges of maximum relative humidity: first, when maximum relative humidity increased from 15% to 45%, and then when maximum relative humidity increased from 60% to 70%. Additionally, they showed that an increase in daily wind speed was associated with a decrease in the number of daily dengue cases. Another study conducted in Sudan showed that relative humidity, precipitation, and maximum and minimum temperatures were correlated with dengue incidence [69]. They found that a 3–5-month lag in relative humidity was the strongest explanatory variable for dengue incidence. This suggests that while temperature, relative humidity, and precipitation are critical factors in understanding and predicting dengue outbreaks, their use should be tailored to specific geographical locations.

### Climatic and environmental factors for mosquito models

Five studies on mosquito models [52,57,61,63,64] utilized temperature and precipitation data in various formats, referred to as bioclimatic variables [70]. These variables included annual mean temperature (BIO1), mean diurnal range (BIO2), isothermality (BIO3), temperature seasonality (BIO4), maximum temperature of the warmest month (BIO5), minimum temperature of the coldest month (BIO6), temperature annual range (BIO7), mean temperature of the wettest quarter (BIO8), mean temperature of the driest quarter (BIO9), mean temperature of the warmest quarter (BIO10), mean temperature of the coldest quarter (BIO11), annual precipitation (BIO12), precipitation of the wettest month (BIO13), precipitation of the driest month (BIO14), precipitation seasonality (BIO15), precipitation of the wettest quarter (BIO16), precipitation of the driest quarter (BIO17), precipitation of the warmest quarter (BIO18), and precipitation of the coldest quarter (BIO19). These bioclimatic variables were primarily used to predict the potential distributions of *Aedes* species under present-day and future climate conditions.

Variables related to habitat/environment were only considered for mosquito models. The most utilized variables were mosquitoes collection location (n = 6), vegetation index (n = 6), followed by habitat type/count (n = 4). Six studies specifically investigated mosquito breeding site locations, with two revealing a higher density of *Ae*. *aegypti* mosquitoes in urban and peri-urban areas compared to rural areas [71,72]. Furthermore, one study indicated that, *Ae*. *albopictus* were more prone in urban and peri-urban areas, whereas *Ae*. *aegypti* were more prevalent in rural areas [73]; meanwhile, another study highlighted the widespread abundance of *Ae*. *aegypti* mosquitoes across both urban and rural settings [56]. Four studies identified a positive significant association between the presence of surrounding vegetation and the presence/abundance of mature/immature *Ae*. *aegypti* and *Ae*. *albopictus* species mosquitoes [50,56,72,74]. Conversely, high habitat counts were observed to significantly contribute to the increased density of *Aedes* mosquitoes [50,55].

## Demographic and socioeconomic factors

Studies focused on determining the rate of dengue infections or prevalence primarily concentrated on demographic and socioeconomic factors. Among the demographic factors, age (n = 6, 20.0%) and gender (n = 6, 20.0%) were frequently examined. In most studies, there was no significant difference in dengue virus infection rates or cases between genders [53,58,59,75–77]. However, some studies did note an association between age and dengue infection, suggesting that children under 5 years of age were less susceptible to dengue virus infection compared to older individuals [58,77]. Additionally, two studies indicated that individuals who travelled outside the country were more prone to dengue virus infection than non-travellers [53,77].

Regarding socioeconomic factors, the type of housing or construction materials used (n = 6, 20.0%) was frequently examined, followed by mosquito preventive measures and household density, each considered by four studies (13.3%). Poor housing conditions were associated with high mosquito density and dengue infection [50,53,54,58,76–78], while overcrowded households were associated with an increased risk of dengue infections [54,76]. Two studies found no significant difference in dengue infection rates between individuals using mosquito bed nets or repellents and those who did not [58,78]. However, one study suggested that not using daily mosquito repellent was associated with an increased risk of dengue infection [53], and another study indicated that respondents using Insecticide-Treated Nets (ITNs) were more likely to be infected with dengue than those who did not use them [77]. Education level and occupation or employment status were also explored as socioeconomic factors, each examined by three studies (10.0%). Some studies did not find a significant association between education level and the risk of dengue infection [58,59,76,77]. However, one study did find that a lack of knowledge about dengue disease was associated with dengue infection within the population [76].

## Larval/Pupal abundance factors

Studies on mosquito models which aimed at assessing the magnitude and geographic dispersion of vector populations have predominantly focused on employing various larval/pupal abundance factors to analyse and forecast the risk and spread of dengue and/or its vector *Aedes* mosquitoes. None of the studies on dengue cases models considered any of the larval/pupal abundance factors in their models. Among the studies reviewed on mosquito models, five included different larval/pupal abundance factors in their analyses. The Breteau Index (BI), indicating *Aedes* larvae-positive containers per 100 houses surveyed, the House Index (HI), reflecting the proportion of infested houses with larvae or pupae, and the Container Index (CI), showing the percentage of water-holding containers with active immature larvae, were each incorporated as factors in the models in four studies [55,71–74]. Other entomological variables like the Pupae Index (PI) [55,72,74], where pupae are counted, the collection location [56,71], and the Pupae per Person Index (PPI) [72] were also considered in some studies.

### Modeling techniques

**Fig 3:**
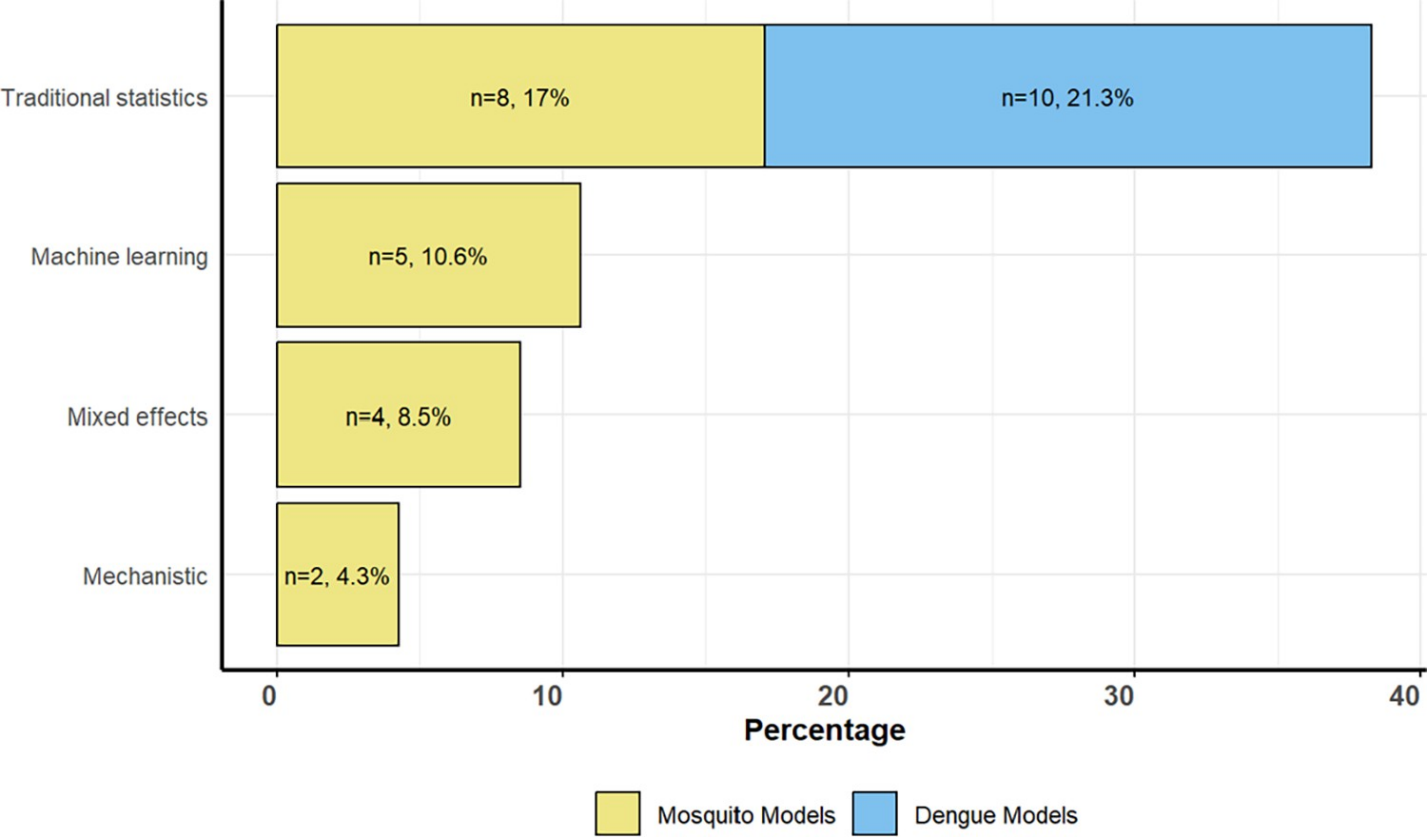
Quantitative model techniques: Mosquito models were found to encompass all modelling techniques, whereas dengue models included only the more traditional statistical techniques.

Various quantitative models were applied to analyse dengue burden, outbreaks, or the presence/absence or abundance of *Aedes* species (for all details see **S2 Table**). The selection of modelling techniques was driven by the specific study objectives, whether they aimed at prediction/forecasting, analysis, or developing early warning systems for dengue and/or *Aedes* vector monitoring. These models were categorized into four classes: machine learning, mechanistic, mixed-effects models, and traditional statistical models (**Fig** 3). Traditional statistical models include common techniques like logistic or linear regression, which (up to a transformation) analyse a linear relationship between variables but do not account for additional complex structures like grouped data or random effects. Mixed-effects models are an advanced form of traditional statistical models, designed to handle data with both fixed and random effects, making them particularly useful for analysing hierarchical or grouped data by considering variability within and between groups. Mechanistic models are based on the biological and physical processes that drive disease transmission or mosquito dynamics. They use mathematical equations to represent how factors like temperature or precipitation influence mosquito populations and dengue transmission, offering a detailed understanding of the underlying mechanisms. Lastly, machine learning models are data-driven approaches that use algorithms to identify patterns in data without being explicitly programmed. These models excel in making predictions and handling large datasets, often providing higher accuracy than traditional methods, particularly in dealing with complex, non-linear relationships.

Mosquito models utilized various classes of these modelling techniques, whereas dengue models relied primarily on traditional statistical models. The predominant modelling approach employed was traditional statistical models (n = 18, 60.0%, including 10 dengue cases models and 8 mosquito models), with logistic regression (binary and multivariable) being the most prevalent (n = 8), followed by generalized linear models (GLMs) (n = 4) and linear regression models (n = 2). One study each utilized Bayesian hierarchical Poisson model (68), Poisson discrete probability (65), generalized additive models [67], and zero-inflated negative binomial models (ZINBs) [49]. Additionally, one study stated that they applied bivariate and multivariate analyses but did not specify the model distribution [77].

Machine learning methodologies were used by 5 studies (16.7%), with 13.3% utilizing maximum entropy (MaxEnt) and 3.3% employing boosted regression tree (BRT) techniques. These methods were commonly employed during the development of mosquito ecological niche or species distribution models (SDM). Their primary goal was to describe the environmental suitability of *Aedes* mosquitoes, especially on larger geographical scales like multicounty or multicontinental levels.

Mixed effect models were employed by 4 studies (13.3%), with generalised linear mixed models (GLMM) being the most prevalent, utilized by 2 studies [56,71]. Generalised additive mixed models (GAMM) (50) and zero-inflated negative binomial mixed effect models (ZINBMs) [55] were each used by one study. Studies often opt for the mixed effect models over traditional statistical model when dealing with nested or clustered data, where observations are grouped within larger units. By incorporating random effects for these groupings, mixed effect models can capture the variability within and between groups more accurately than traditional models.

Mechanistic models were employed by 2 studies (6.7%), whereas one study used general circulation models (GCMs) to predict monthly dengue global transmission risk in current climates and compare it to expected risk in 2050 and 2080 on a global scale [62]. Another study employed the similarity search approach to create a risk map by integrating environmental susceptibility analysis and geographical information systems [52]. It then compared areas with dengue prevalence to all other locations.

### Variable selection/Dimension reduction

In this review, a total of 11 (36.7%) studies explicitly stated the techniques they used for variable selection/dimension reduction during their model development (see **S2 Table** for details). These techniques conveyed significant diversity, with the most frequently used technique being the Akaike Information Criterion (AIC), which was employed by 6 studies, making up 20.0% of all the articles reviewed. The second most common approach was Principal Component Analysis (PCA) [61] and Multi Correspondence Analysis (MCA) [54], implemented by 2 studies and accounting for 6.7% of the reviewed articles. Stepwise procedures [75], Unbiased Risk Estimator (UBRE) [67], likelihood ratio tests [58], Jackknife procedures [57], and Bayesian Information Criterion (BIC) [50] were each used by 1 study.

### Model validation or predictive performance

In the 30 studies reviewed, only six (20.0%) specified the validation techniques used to assess model performance (**S2 Table**). Among these techniques, confusion matrix-based metrics with partial receiver operating characteristic (pROC) curve were the most employed technique, implemented by 4 (13.3%) studies. The second most utilized technique for model validation was the Area Under the Curve (AUC), used by 3 (10.33%) studies. Other commonly employed validation techniques included analysis of residual plots and partitioning the dataset into training and test sets (cross-validation).

## Discussion

This comprehensive review explores the quantitative models used for modelling and predicting the spread/outbreak of dengue cases/incidences/prevalence or the abundance/presence of its vector, *Aedes* mosquitoes, in the context of Africa.

The nature of data, including model outcomes and covariates, plays a significant role in determining the type and structure of models within the field of modelling. Notably, two-thirds of the studies used were “mosquito models” aiming at to predicting or modelling the population dynamics of dengue vectors as a proxy risk factor for dengue. These studies focus on monitoring *Aedes* mosquito populations and their breeding sites, serving as early indicators of potential dengue outbreaks by identifying areas with high vector densities. While this proactive approach is essential for implementing targeted vector control measures and can help prevent outbreaks in human populations, it is important to note that the majority (65%) of these mosquito data sets were obtained via entomological surveys, that can come with significant associated costs and resource demands. Conducting regular entomological surveys to gather essential data for dengue modelling and Early Warning System (EWS) development requires a significant financial investment. These include expenses related to personnel, equipment, fieldwork logistics, data collection, and subsequent analysis.

One third of the identified studies, utilized human dengue data as a model outcome and encompass parameters like the number of confirmed dengue cases [54,65,67,69], individuals with active dengue infections [53,58,75,77,78], and those testing seropositive for dengue IgG and IgM antibodies [59,76]. Employing human dengue cases as an indicator for detecting dengue outbreaks provides a direct measure of dengue transmission and its impact on the susceptible population. This approach can be particularly beneficial in areas where implementing comprehensive entomological surveillance is challenging. However, it is crucial to ensure the accuracy and timely availability of dengue cases data. This necessitates reliance on reliable and high-quality data sources such as government surveillance systems, including hospitals, and other trustworthy sources. Despite the advantages of using government surveillance systems to obtain accurate dengue cases data, only a small proportion of the identified studies (n = 6) adopted this approach. Instead, about half of the human dengue modelling studies in Africa (n = 20) relied on cross-sectional surveys, which can incur significant costs during the data collection process, and which may therefore not form a sustainable backbone in a dengue early warning system for a country. This pattern contrasts with trends observed in other parts of the world, where more than two-thirds of the dengue cases data in reviewed studies came from surveillance systems [33,45]. The preference of utilizing cross-sectional human dengue or entomological surveys in Africa may be attributed by the challenges of unavailable dengue surveillance systems [79], leading to underreporting or poor-quality data that complicates the development of accurate models.

It was observed that 60% of the all the identified studies utilized traditional statistical (correlative) models, with logistic regression being the most used approach, this is consistent with the results of another systematic review [33]. Traditional statistical regression models work under several assumptions, including linearity (meaning the relationship between the dependent variable and the explanatory variables is assumed to be linear) and homogeneity of variance (indicating that the variability of the residuals is constant across all levels of the explanatory variables). However, it is acknowledged that most of these assumptions may not always reflect real-world scenarios accurately, especially when dealing with complex relationships between variables like climatic factors, which can be non-linear [80,81]. Moreover, when trying to forecast disease outbreaks over time and in specific regions, traditional statistical regression techniques may face difficulties. To address these challenges, researchers can consider using non-parametric models or adding random effects to their models [82,83]. These adjustments aim to enhance the model’s flexibility and its ability to capture nuanced relationships within the data. In studies conducted in Africa, only a few studies (13.3%) have adopted these advanced techniques, such as generalized linear mixed models (GLMM), generalized additive mixed models (GAMM), and zero-inflated negative binomial mixed models (ZINBMs).

Regarding spatio-temporal models, which integrate geographical and temporal aspects, none of the identified studies conducted in Africa considered these types of models. Spatial models account for the geographical distribution and clustering of disease cases, as well as correlations between spatial sampling units, while temporal models capture the patterns and trends of dengue occurrence over time. Spatio-temporal models, on the other hand, integrate both dimensions, providing a holistic view of disease spread across different locations and time intervals. These models can offer valuable insights for risk assessments, aiding local or national dengue prevention and control programs in preparing for and responding to dengue epidemics in endemic regions. For instance, Patricia Marques et al. [84] used a Bayesian hierarchical framework to forecast dengue dynamics in Brazil, revealing that some traditionally non-endemic microregions might experience increased dengue incidence due to future climate scenarios. Similarly, Hwa-Lung Yu et al. [85] employed a spatio-temporal approach to predict dengue outbreaks in Southern Taiwan, highlighting the significant impact of climatic conditions and providing valuable "one-week-ahead" outbreak warnings. Moreover, in Singapore, Haoyang Sun et al. [86] utilized a Bayesian hierarchical model to analyse the spatio-temporal dynamics of *Aedes aegypti* and *Aedes albopictus* in relation to environmental and anthropogenic variables. Their findings suggested that public residential estates with older buildings and more nearby managed vegetation should be prioritized for vector control inspections and community advocacy to reduce *Aedes* mosquito abundance and mitigate dengue risk. Dengue is sensitive to variations in climatic conditions at local, regional, and global scales. Some areas currently at risk, but not yet endemic for dengue, may transition to endemic status due to climate change, particularly related to temperature changes. The absence of these models in Africa may stem from various reasons, notably the insufficient capacity and the unavailability of suitable data required for their construction. This may be due to the lack of a dengue surveillance data system. The quality of accessible data, particularly surveillance data, directly influences the caliber of models that can be created. Inadequate and inaccurate data pose significant challenges to developing effective spatio-temporal models.

In many studies across several African countries, like findings in other parts of the world, certain environmental factors such as rainfall, temperature, and relative humidity consistently emerged as crucial covariates influencing the transmission of dengue and the presence or abundance of its vector, *Aedes* mosquitoes. These climatic variables play a significant role in the breeding and survival of *Aedes* mosquitoes, consequently impacting the prevalence and spread of dengue. However, beyond these well-established factors, several other significant variables affecting the spread of dengue in Africa were also observed. One such factor is the presence of surrounding vegetation, which has been notably associated with the presence of immature stages of *Ae*. *aegypti* and *Ae*. *albopictus*. Vegetation provides ideal breeding grounds for these mosquitoes, leading to increased transmission rates in areas with dense vegetation. Additionally, poor housing conditions have emerged as strong indicators of higher dengue incidence in African settings. Other socio-economic factors, such as lack of public knowledge about dengue and overcrowded households, were also found to be influential determinants of dengue risk. While vector abundance is a well-recognized risk factor for dengue outbreaks, none of the analysed studies incorporated these entomological indicators directly into their models. This omission is particularly surprising, as mosquito abundance is a key driver of dengue transmission. Including such indicators in future models could improve the accuracy and effectiveness of outbreak predictions in Africa.

Evaluation metrics play a critical role in real-world data studies as they assess whether the collected data suit the models’ intended objectives and help gauge data quality and bias [87,88]. Our findings uncovered a concerning trend regarding the absence of robust variable selection procedures and model validation among the reviewed published models. Many predictive models heavily depend on substantial data for accurate disease modelling. Therefore, steps like variable selection and model validation are vital to counter overfitting and enhance the interpretability and predictive accuracy of these models. Variable selection is pivotal as it involves identifying and including the most relevant covariates from a larger set to create a parsimonious model. This process is crucial because it eliminates extraneous or redundant variables, thus reducing overfitting—where a model becomes too tailored on training data and performs poorly on test data—while also improving the model’s interpretability, making it more understandable and applicable in real-world scenarios. Additionally, model validation is imperative to ensure the reliability and accuracy of predictive models. Validation should not only be conducted within the population from which the data were sourced but also across diverse populations to assess generalizability. It is concerning that our review identified a lack of explicit use of out-of-sample validation techniques in the reviewed studies, indicating a potential gap in ensuring the robustness and applicability of these models beyond their training data.

### Towards sustainable early warning systems for dengue in Africa

Having an effective EWS for dengue is important for controlling the disease before it becomes a big problem. This works by using data from human case surveillance, monitoring mosquito abundances, looking at environmental information, and using advanced modelling techniques to predict and spot outbreaks before they become serious public health issues [89,90]. For a model to serve as an effective EWS for dengue, it must have the capability to forecast disease outbreaks proactively, identify areas or populations at high risk of infection, and integrate both spatial and temporal aspects. Such a model should integrate real-time data, including climate conditions, mosquito population dynamics, and historical disease data, to deliver timely and precise predictions. In the studies reviewed, none had specifically developed models tailored for dengue early warning systems in the context of Africa. This gap can be traced back to the limited and inconsistent availability of data. This scarcity is a direct result of the absence of comprehensive dengue surveillance systems in several African countries. As a result, many existing models lean towards being descriptive rather than predictive. They are primarily focused on comprehending the present distributions of dengue and/or *Aedes* species, as well as their influencing factors, rather than anticipating and forecasting future outbreaks. As a way forward, we suggest the following recommendations, addressing the gap of dengue EWS in Africa:

✓ African governing bodies must prioritize enhancing dengue surveillance systems to address the scarcity of reliable and high-quality dengue data. This can be accomplished by expanding existing surveillance efforts from other diseases, like those conducted by National Malaria Control Programmes, to also include dengue information. If no such systems are currently in place, existing resources can be utilized to introduce a cost-effective surveillance system. By fostering improved surveillance systems, we can achieve more accurate and comprehensive data collection, which is essential for developing effective dengue models. This, in turn, will enable the creation of EWS for disease outbreaks, providing timely alerts and facilitating proactive measures to mitigate the impact of dengue on public health.
✓ Ongoing entomological surveys in Africa are essential for modelling dengue and *Aedes* mosquitoes and developing EWS. This is especially important given the limited dengue surveillance currently available on the continent. However, these surveys need to be carefully designed or refined to collect the appropriate data necessary for building an effective predictive model. To ensure the effectiveness and sustainability of these surveys, essential support from key stakeholders such as governing bodies, public health organizations, and research institutions is inevitable. This collaborative effort will not only enhance data collection but also contribute to the development of accurate and appropriate predictive models, thereby improving the effectiveness of dengue EWS and *Aedes* mosquito-related diseases.
✓ An effective predictive model that incorporates covariates from multiple domains, including climatic, environmental, demographic, socioeconomic, and larval/pupal abundance factors, would be highly advantageous. However, it is crucial to test whether the inclusion of any of these covariates improves the model’s predictive capabilities.

### Limitations

Our analysis was limited to studies published in English, which may have impacted our evaluation of regional trends. Furthermore, there is a possibility that relevant literature, including some grey literature, were not included as databases do not cover all journals and university press articles. This is particularly crucial for locally significant modelling efforts that may not have reached mainstream academic platforms.

## Conclusion

We conducted a comprehensive systematic review, specifically examining the quantitative methods used to model and/or predict dengue or its vector *Aedes* mosquito in Africa. Our review identified several key shortcomings in the current modelling practices for dengue in Africa, including scarce dengue surveillance systems, inadequate reporting of model development techniques, validation, and performance measures. We also observed the predominant focus on traditional statistical methodology in modelling techniques, with a lack of utilization of more advanced models such as spatio-temporal models–crucial to enable real-time prediction. Additionally, our review highlighted a lack of adoption of models suitable for serving as dengue Early Warning Systems (EWS) in Africa. We hope the findings of this review will aid in paving the way for improving dengue modelling practices as dengue continues to spread/increase across the continent, making the development and implementation of appropriate dengue EWS are more critical than ever.

## Supporting information

S1 PRISMA Checklist PRISMA 2020 checklist. (DOCX)

S1 Table Databases and search terms. (DOCX)

S2 Table Characteristics of the included quantitative models. (DOCX)
