# Supplementary material for: Quantitative modelling for dengue and Aedes mosquitoes in Africa: A systematic review of current approaches and future directions for Early Warning System development
Source: PLoS Negl Trop Dis. 2024 Nov 26;18(11):e0012679. doi: 10.1371/journal.pntd.0012679 (PMC11630623; doi:10.1371/journal.pntd.0012679)
Supplement: S1 Table — (DOCX) [file pntd.0012679.s002.docx]

**Table:** Databases and search terms

| **Database** | **Keywords** | **Identified** | **Retrieved** |
| --- | --- | --- | --- |
| PubMed | ((model* OR analysis OR forecast* OR predict* OR interpolation OR projection OR mapping) AND (dengue OR dengue fever OR dengue outbreak OR dengue virus OR dengue haemorrhagic fever OR DHF OR dengue shock syndrome OR Aedes aegypti OR Aedes albopictus OR Aedes mosquitoes OR vector abundance OR dengue*)) AND (Algeria OR Angola OR Benin OR Botswana OR Burkina Faso OR Burundi OR Cabo Verde OR Cameroon OR Central African Republic OR Chad OR Comoros OR Congo OR Cote* OR Djibouti OR Egypt OR Equatorial Guinea OR Eritrea OR Eswatini OR Ethiopia OR Gabon OR Gambia OR Ghana OR Guinea OR Guinea-Bissau OR Kenya OR Lesotho OR Liberia OR Libya OR Madagascar OR Malawi OR Mali OR Mauritania OR Mauritius OR Morocco OR Mozambique OR Namibia OR Niger OR Nigeria OR Rwanda OR Sao Tome and Principe OR Senegal OR Seychelles OR Sierra Leone OR Somalia OR South Africa OR South Sudan OR Sudan OR Tanzania OR Togo OR Tunisia OR Uganda OR Zambia OR Zanzibar OR Zimbabwe OR Africa*) | 2,779 | 54 |
| EMBASE | ((model* OR analysis OR forecast* OR predict* OR interpolation OR projection OR mapping) AND (dengue OR dengue fever OR dengue outbreak OR dengue virus OR dengue haemorrhagic fever OR DHF OR dengue shock syndrome OR Aedes aegypti OR Aedes albopictus OR Aedes mosquitoes OR vector abundance OR dengue*)) AND (Algeria OR Angola OR Benin OR Botswana OR Burkina Faso OR Burundi OR Cabo Verde OR Cameroon OR Central African Republic OR Chad OR Comoros OR Congo OR Cote* OR Djibouti OR Egypt OR Equatorial Guinea OR Eritrea OR Eswatini OR Ethiopia OR Gabon OR Gambia OR Ghana OR Guinea OR Guinea-Bissau OR Kenya OR Lesotho OR Liberia OR Libya OR Madagascar OR Malawi OR Mali OR Mauritania OR Mauritius OR Morocco OR Mozambique OR Namibia OR Niger OR Nigeria OR Rwanda OR Sao Tome and Principe OR Senegal OR Seychelles OR Sierra Leone OR Somalia OR South Africa OR South Sudan OR Sudan OR Tanzania OR Togo OR Tunisia OR Uganda OR Zambia OR Zanzibar OR Zimbabwe OR Africa*) | 2,449 | 43 |
| EBSCOhost | ((model* OR analysis OR forecast* OR predict* OR interpolation OR projection OR mapping) AND (dengue OR dengue fever OR dengue outbreak OR dengue virus OR dengue haemorrhagic fever OR DHF OR dengue shock syndrome OR Aedes aegypti OR Aedes albopictus OR Aedes mosquitoes OR vector abundance OR dengue*)) AND (Algeria OR Angola OR Benin OR Botswana OR Burkina Faso OR Burundi OR Cabo Verde OR Cameroon OR Central African Republic OR Chad OR Comoros OR Congo OR Cote* OR Djibouti OR Egypt OR Equatorial Guinea OR Eritrea OR Eswatini OR Ethiopia OR Gabon OR Gambia OR Ghana OR Guinea OR Guinea-Bissau OR Kenya OR Lesotho OR Liberia OR Libya OR Madagascar OR Malawi OR Mali OR Mauritania OR Mauritius OR Morocco OR Mozambique OR Namibia OR Niger OR Nigeria OR Rwanda OR Sao Tome and Principe OR Senegal OR Seychelles OR Sierra Leone OR Somalia OR South Africa OR South Sudan OR Sudan OR Tanzania OR Togo OR Tunisia OR Uganda OR Zambia OR Zanzibar OR Zimbabwe OR Africa*) | 1,080 | 44 |
| SCOPUS | "model*" OR "mapping" OR "analysis" OR "forecast*" OR "predict*" OR “interpolation” OR “projection”AND "dengue" OR "dengue fever" OR "dengue virus" OR "dengue haemorrhagic fever" OR "DHF" OR "dengue shock syndrome" OR "Aedes aegypti" OR "Aedes albopictus" | 1,029 | 37 |
