# Supplementary material for: Quantitative modelling for dengue and Aedes mosquitoes in Africa: A systematic review of current approaches and future directions for Early Warning System development
Source: PLoS Negl Trop Dis. 2024 Nov 26;18(11):e0012679. doi: 10.1371/journal.pntd.0012679 (PMC11630623; doi:10.1371/journal.pntd.0012679)
Supplement: S2 Table — (DOCX) [file pntd.0012679.s003.docx]

**S2 Table:** Characteristics of the included quantitative models

| **Author and publication Year** | **Country** | **Model outcome** | | **Dengue and/or Aedes data sources** | **Geographical scope** | **Covariates** | | | **Data sources** | **Quantitative Analysis Method** | **Variable Selection/Dimension Reduction** | **Model Validation** |
| --- | --- | --- | --- | --- | --- | --- | --- | --- | --- | --- | --- | --- |
|  |  | **Dengue models** | **Mosquito models** |  |  | **Environmental/Climatic** | **Ecological** | **Demographic and Socio-economic factors** |  |  |  |  |
| Khan A et al. (2023) (1) | Kenya | Laboratory confirmed number of dengue |  | Longitudinal survey from 2014 - 2018 | Regional level (Western and Coastal of Kenya) |  |  | Window screens, bennet ownership, crowed household, metal roof, litter presence | Survey | Poisson (GLMs) | Multi Correspondence Analysis (MCA) and Akaike Information Criterion (AIC) |  |
| Mwakutwaa AS et al. (2023) (2) | Kenya |  | Number of *Ae. aegypti* pupae | Entomological surveys from June 2 to June 17, 2017 | Town (Bomani) | Habitat type |  |  | Survey | Zero inflated negative binominal regression |  |  |
| Ouattara CA et al. (2022) (3) | Burkina Faso | Monthly dengue cases (lab-confirmed) |  | MOH - Health statistical yearbook | District level |  |  |  |  | Poisson Discrete probability |  |  |
| Badolo A et al. (2022) (4) | Burkina Faso |  | *Ae. aegypti* adult mosquito abundance, number of *Aedes aegypti* larvae | Longitudinal Survey from 2016 - 2017 | District level | Temperature (minimal, maximal, and mean), relative humidity and daily rainfall | House index (HI), Container index (CI), Breteau index (BI), Locality (urban, peri-urban, rural), collection location (indoors, outdoors), |  | National Meteorological Agency | Generalised Linear Mixed Models (GLMMs) with negative binomial link | Akaike Information Criterion (AIC) |  |
| Owusu-Asenso CM et al. (2022) (5) | Ghana |  | Abundance of *Aedes* larvae | Entomological surveys from 2017 - 2018 | Zones | Seasonal (Dry, Rainy), Ecological zones (Coastal savannah, Forest, Sahel savannah) |  | Population (Urban, Suburban, Rural) |  | Generalized linear model (GLMs) analysis |  |  |
| Ouattara C.A. et al. (2022) (6) | Burkina Faso | Total weekly dengue cases (lab-confirmed) |  | MOH - National epidemiological surveillance system | District level | Weekly rainfall, weekly mean of daily minimum and maximum temperature, weekly mean of daily minimum and maximum relative humidity, and weekly mean of daily average wind speed |  |  | National Aeronautics and Space Administration’s Earth Observing System data and Information System | Generalized additive model (GAMs) with a negative binomial distribution | Unbiased risk estimator (UBRE) |  |
| Abdelkrim O et al. (2021) (7) | Morocco |  | Presence (*Aedes* species (*Ae. Aegypti, Ae. Albopictus, Ae. vexans, Ae. vittatus, Ae. caspius* and *Ae. detritus*)) | Culicidae Morocco database responsible for tracing back the history of mosquitos and from Global Biodiversity Information Facility | Regional level | Bioclimatic variables derived from monthly temperature and rainfall, altitude |  |  | WorldClim | Machine-learning (maximum entropy principle) | Akaike Information Criterion (AIC) | Split the data into training and testing sets and pROC and AUC |
| Lim JK et al. (2021) (8) | Gabon | Laboratory confirmed dengue infection status |  | Surveillance study from May 2015 to December 2016 in Hospital (Albert Schweitzer Hospital) | City (Lambare´ne´) |  |  | Patient age and gender, body temperature at enrolment, fever duration prior to visit, yellow fever vaccination and presence of signs and symptoms | Survey | Logistic regression | Backward stepwise process |  |
| Kampango A et al. (2021) (9) | Zanzibar |  | Number of *Ae. aegypti* and *Ae. bromeliae* | Entomological surveys | Region (south-eastern costal region) | Seasonal (Dry, Rainy), Sun exposure (Half a day, more than half a day), Vegetation (Yes/No),  Type of habitant and Habitant size (small, medium, large) | Container index (CI), pupae per container (PPC) and proportion of positive containers (PC) |  | Survey | Zero inflated negative binomial mixed models (ZINBMs) |  |  |
| Djiappi-Tchamen B et al. (2021) (10) | Cameroon |  | *Aedes* species abundance *(Ae. albopictus, Ae.*  *aegytpi, Ae. simpsoni* and *Aedes spp.)* | Entomological survey conducted from November 2019 to November 2020 | City (Yaoundé city) | Seasonal (dry/rainy),  Breeding Habitats | Breteau Index (BI), Container index (CI), House index (HI), Locality (urban, peri-urban, rural) |  | Survey | Generalized Linear Models (GLMs) |  |  |
| Omar K et al. (2021) (11) | Nigeria |  | Number of *Aedes* mosquitoes | Entomological survey conducted between November 2017 and September 2018 | State (Enugu) | Bioclimatic variables derived from monthly temperature and rainfall, elevation |  |  | WorldClim and Shuttle Radar Topography Mission (SRTM) | Machine-learning (maximum entropy principle) |  | Area under the curve (AUC) and partial receiver operating characteristic (pROC) |
| Elaagip A et al. (2020) (12) | Sudan | Dengue prevalence |  | Cross-sectional community-based study was conducted from March 2016 to March 2017 and entomological survey | State (Kassala) |  |  | House type, construction materials, and household density, Occupation | Survey | Multiple logistic regression model |  |  |
| Saleh F et al. (2020) (13) | Zanzibar |  | Presence/absence of *Ae. aegypti* (immature or pupae), number of *Ae. aegypti* (immature or pupae) | Cross-sectional entomological survey conducted from February to May 2018 | District level (Urban District and the West District) of Unguja region | Seasonal (Dry, Rainy), Sun exposure (Half a day, more than half a day), Vegetation (Yes/No) | Function (water storage, discarded objects, other) Water volume (low, medium, high), collection location (indoors, outdoors) | Setting (urban, rural) | Survey | Generalized linear mixed models (GLMMs) | Akaike Information Criterion (AIC) |  |
| Ngugi et al. (2020) (14) | Kenya |  | Monthly number of *Aedes aegypti* pupae | Entomological and demographic surveys conducted from July 2014 to June 2018 | Region (coastal and inland) | Temperature and total rainfall, habitat count, and bushes/tall grass |  | House wall material, House roof material, No. of rooms, No. of sleepers, Insecticide/coil use, Bed net use, Firewood use, Eaves open, and Room ceilings | Data from two installed temperature loggers, local metrological weather data, and National Oceanic and Atmospheric Administration | Generalized additive mixed model (GAMMs) | Akaike Information Criterion (AIC) and Bayesian Information Criterion (BIC) | Residual analysis |
| Wilson-Bahun et al. (2020) (15) | Republic of the Congo |  | Presence of immature stages (larvae and pupae) or pupae only of each *Aedes* species and number of immature stages and pupae only of *Aedes spp.* | Entomological survey carried out in November/December 2017 | City (Brazzaville) | Vegetation (Yes/No), Distance of larval habitant to nearest building (0-5m, 5-10m) | Breteau index (BI), house index (HI), container index (CI), pupae index (PI) and pupae per person index (PPI), Locality (urban, peri-urban, rural), | Container types, Water quality (clear, turbid, polluted), Water source (tap, rain) | Survey | Binary logistic regression model and negative binomial regression model | Akaike Information Criterion (AIC) |  |
| Noureldin E et al. (2019) (16) | Sudan | Monthly number of dengue fever/dengue haemorrhagic fever |  | MOH - Department of Epidemiology. | Port Sudan | Minimum temperature, maximum temperature, relative humidity, and rainfall |  |  | Sudan Meteorological Authority | Multiple linear regression |  |  |
| Iyaloo et al. (2019) (17) | Mauritius |  | Ovitrap positivity (*Ae. albopictus*) | Entomological survey was carried out from February 2013 to May 2015 | Villages | Monthly mean temperature, monthly mean relative humidity, and monthly cumulative rainfall |  |  | HOBO weather data logger and Mauritius Meteorological Services | Multiple regression |  |  |
| Leta S et al. (2018) (18) | Multicontinental |  | Number of countries affected with arboviral diseases (including dengue) and Number of countries/territories suitable for *Aedes aegypti* and *Aedes albopictus* | Secondary data | Multicontinental |  |  |  |  | Machine learning (Boosted regression trees (BRT) model) |  |  |
| Kamal M et al. (2018) (19) | Multicontinental |  | Occurrence records (*Ae. aegypti* and *Ae. albopictus*) | Secondary data (From published studies) | Multicontinental | Bioclimatic variables derived from monthly temperature and rainfall |  |  | WorldClim | Machine-learning (maximum entropy principle) | Principal Component Analysis (PCA) | Split the data into training and testing sets and partial receiver operating characteristic (pROC) |
| Ryan S.J. et al. (2018) (20) | Multicontinental |  | *Aedes (Ae. aegypti and Ae. albopictus)*-borne viral transmission risk |  | Multicounty | Mean monthly temperature |  | Population count data | WorldClim and Gridded Population of the World, version 4 (GPW4) | General circulation models (GCMs) (Temperature dependent R0 Model) |  |  |
| Camara N. et al. (2018) (21) | Tanzania | Dengue infections |  | Cross sectional survey | District level |  |  | Gender, age(years), employment, type of employment, residence, no. household members, use of mosquito repellents, screen windows | Cross sectional survey | Multivariable logistic regression | Likelihood ratio test |  |
| Kajeguka D.C. et al. (2017) (22) | Tanzania | Dengue IgM seropositivity |  | Cross sectional survey | District level |  | Locality (urban, peri-urban, rural), | Age, gender, level of education, symptoms | Cross sectional survey | Logistic regression |  |  |
| Agha et al. (2017) (23) | Kenya |  | Total mosquito abundance and specific-species abundance (*Ae. aegypti* and *Ae. bromeliae*) | Entomological surveys | Cities (Kilifi, Nairobi and Kisumu) |  |  |  |  | General linear models (GLMs) |  | Residual analysis |
| Mweya C.N. et al. (2016) (24) | Tanzania |  | Infected *Aedes aegypti* | Entomological surveys | City (Dar Es Salaam) | Bioclimatic variables derived from monthly temperature and rainfall |  |  | WorldClim and Coupled Model Inter-comparison Project Phase 5 (CMIP5) | Ecological niche models (maximum entropy principle) | Jackknife procedures | Partial receiver operating characteristic (PROC) and area under the curve (AUC) |
| Ellis EM et al. (2015) (25) | Kenya | Dengue infections |  | Hospital surveillance and seroincidence survey | City (Mombasa) |  |  | Gender, age, religion, dengue symptoms, medical outcome, use of mosquito repellents, screen windows, use air conditioning, use mosquito coils, home construction, open windows at night, travel outside the country last 3 months, use bed net, household sick last month, breeding container in yard | Survey | Logistic regression |  |  |
| Soghaier et al. (2015) (26) | Sudan | Dengue seropositive |  | Cross-sectional community-based study | State (Kassala) |  |  | Age, gender, education level, relation of the respondent to household head, residence status |  | Multivariate logistic regression model |  |  |
| Althouse B.M. et al. (2015) (27) | Senegal |  | Yearly mosquito abundance | Routine arbovirus surveillance from Kedougou region | Region | Yearly mean temperature, yearly mean relative humidity, and yearly sum of total rainfall |  |  | National Oceanic and Atmospheric Administration (NOAA) | Bayesian hierarchical Poisson model |  |  |
| Attaway DF et al. (2014) (28) | Kenya |  | Mosquito habitat | MosquitoMap, ProMed, PubMed and Weekly Epidemiological Report from WHO | National level | Bioclimatic variables derived from monthly temperature and rainfall |  | Human population density data | MosquitoMap and LandScan 2011 Global Population Database | Risk maps |  |  |
| Mazaba-Liwewe ML et al. (2014) (29) | Zambia | Dengue infections |  | cross sectional study | Provinces (Western and North-Western) |  |  | Age, gender, occupation, education level, visitation to Angola and/or DRC, use of mosquito preventive measures (ITNs and Insecticide Residual Spray) and type of roofing | Cross-sectional study | Bivariate and multivariate analyses (No model distribution) |  |  |
| Kamgang B et al. (2013) (30) | Central African Republic |  | Number of immature *Ae. albopictus* and *Ae. aegypti* | Entomological surveys | Region (Southern) | Sun exposure, Plant debris inside the container, and Vegetation around the container | House index (HI), Breteau index (BI), larvae index (number of larvae L3-4 per 100 houses), the pupae index (number of pupae per 100 houses), and Type of container | Water quality (clear, turbid, polluted) | Entomological surveys | Binary logistic regression |  |  |

**Reference**

1. Khan A, Bisanzio D, Mutuku F, Ndenga B, Grossi-Soyster EN, Jembe Z, et al. Spatiotemporal overlapping of dengue, chikungunya, and malaria infections in children in Kenya. BMC Infect Dis. 2023 Dec 1;23(1).

2. Mwakutwaa AS, Ngugi HN, Ndenga BA, Krystosik A, Ngari M, Abubakar LU, et al. Pupal productivity of larval habitats of Aedes aegypti in Msambweni, Kwale County, Kenya. Parasitol Res. 2023 Mar 1;122(3):801–14.

3. Ouattara CA, Traore S, Sangare I, Traore TI, Meda ZC, Savadogo LGB. Spatiotemporal analysis of dengue fever in Burkina Faso from 2016 to 2019. BMC Public Health. 2022 Dec 1;22(1).

4. Badolo A, Sombié A, Yaméogo F, Wangrawa DW, Sanon A, Pignatelli PM, et al. First comprehensive analysis of Aedes aegypti bionomics during an arbovirus outbreak in west Africa: Dengue in Ouagadougou, Burkina Faso, 2016–2017. PLoS Negl Trop Dis. 2022;16(7).

5. Owusu-Asenso CM, Mingle JAA, Weetman D, Afrane YA. Spatiotemporal distribution and insecticide resistance status of Aedes aegypti in Ghana. Parasit Vectors. 2022 Dec 1;15(1).

6. Ouattara CA, Traore TI, Traore S, Sangare I, Meda CZ, Savadogo LGB. Climate factors and dengue fever in Burkina Faso from 2017 to 2019. J Public Health Afr. 2022 May 24;13(1).

7. Abdelkrim O, Samia B, Said Z, Souad L. Modeling and mapping the habitat suitability and the potential distribution of Arboviruses vectors in Morocco. Parasite. 2021;28.

8. Lim JK, Fernandes JF, Yoon IK, Lee JS, Mba RO, Lee KS, et al. Epidemiology of dengue fever in gabon: Results from a health facility-based fever surveillance in lambaréné and its surroundings. PLoS Negl Trop Dis. 2021 Feb 1;15(2):1–15.

9. Kampango A, Furu P, Sarath DL, Haji KA, Konradsen F, Schiøler KL, et al. Risk factors for occurrence and abundance of Aedes aegypti and Aedes bromeliae at hotel compounds in Zanzibar. Parasit Vectors. 2021 Dec 1;14(1).

10. Djiappi-Tchamen B, Nana-Ndjangwo MS, Tchuinkam T, Makoudjou I, Nchoutpouen E, Kopya E, et al. Aedes mosquito distribution along a transect from rural to urban settings in Yaoundé, Cameroon. Insects. 2021 Sep 1;12(9).

11. Omar K, Thabet HS, TagEldin RA, Asadu CC, Chukwuekezie OC, Ochu JC, et al. Ecological niche modeling for predicting the potential geographical distribution of Aedes species (Diptera: Culicidae): A case study of Enugu State, Nigeria. Parasite Epidemiol Control. 2021 Nov 1;15.

12. Elaagip A, Alsedig K, Altahir O, Ageep T, Ahmed A, Siam HA, et al. Seroprevalence and associated risk factors of Dengue fever in Kassala state, eastern Sudan. PLoS Negl Trop Dis. 2020 Dec 1;14(12):e0008918.

13. Saleh F, Kitau J, Konradsen F, Kampango A, Abassi R, Schiøler KL. Epidemic risk of arboviral diseases: Determining the habitats, spatial-temporal distribution, and abundance of immature Aedes aegypti in the Urban and Rural areas of Zanzibar, Tanzania. PLoS Negl Trop Dis. 2020 Dec 1;14(12).

14. Ngugi HN, Nyathi S, Krystosik A, Ndenga B, Mbakaya JO, Aswani P, et al. Risk factors for Aedes aegypti household pupal persistence in longitudinal entomological household surveys in urban and rural Kenya. Parasit Vectors. 2020 Oct 1;13(1).

15. Wilson-Bahun TA, Kamgang B, Lenga A, Wondji CS. Larval ecology and infestation indices of two major arbovirus vectors, Aedes aegypti and Aedes albopictus (Diptera: Culicidae), in Brazzaville, the capital city of the Republic of the Congo. Parasit Vectors. 2020 Sep 25;13(1).

16. Noureldin E, Shaffer L. Role of climatic factors in the incidence of dengue in port sudan city, sudan. Eastern Mediterranean Health Journal. 2019;25(12):852–60.

17. Iyaloo DP, Facknath S, Bheecarry A. Seasonal Changes in the Distribution and Relative Abundance of the Dengue Vector, Aedes albopictus in Two Villages in Mauritius: Implications for a Sterile Release Programme. African Entomology. 2019 Mar 1;27(1):218–27.

18. Leta S, Beyene TJ, De Clercq EM, Amenu K, Kraemer MUG, Revie CW. Global risk mapping for major diseases transmitted by Aedes aegypti and Aedes albopictus. International Journal of Infectious Diseases. 2018 Feb 1;67:25–35.

19. Kamal M, Kenawy MA, Rady MH, Khaled AS, Samy AM. Mapping the global potential distributions of two arboviral vectors Aedes aegypti and Ae. Albopictus under changing climate. PLoS One. 2018 Dec 1;13(12).

20. Ryan SJ, Carlson CJ, Mordecai EA, Johnson LR. Global expansion and redistribution of Aedes-borne virus transmission risk with climate change. PLoS Negl Trop Dis. 2018 Mar 1;13(3).

21. Camara N, Ngasala B, Leyna G, Abade A, Rumisha SF, Oriyo NM, et al. Socio-demographic determinants of dengue infection during an outbreak in Dar es Salaam city, Tanzania. Tanzan J Health Res. 2018;20(2).

22. Kajeguka DC, Kaaya RD, Desrochers R, Iranpour M, Kavishe RA, Mwakalinga S, et al. Mapping clusters of chikungunya and dengue transmission in northern Tanzania using disease exposure and vector data. Tanzan J Health Res. 2017 Oct 1;19(4).

23. Agha SB, Tchouassi DP, Bastos ADS, Sang R. Dengue and yellow fever virus vectors: Seasonal abundance, diversity and resting preferences in three Kenyan cities. Parasit Vectors. 2017 Dec 29;10(1).

24. Mweya CN, Kimera SI, Stanley G, Misinzo G, Mboera LEG. Climate change influences potential distribution of infected Aedes aegypti co-occurrence with dengue epidemics risk areas in Tanzania. PLoS One. 2016 Sep 1;11(9).

25. Ellis EM, Neatherlin JC, Delorey M, Ochieng M, Mohamed AH, Mogeni DO, et al. A Household Serosurvey to Estimate the Magnitude of a Dengue Outbreak in Mombasa, Kenya, 2013. PLoS Negl Trop Dis. 2015 Apr 29;9(4).

26. Soghaier MA, Himatt S, Osman KE, Okoued SI, Seidahmed OE, Beatty ME, et al. Cross-sectional community-based study of the socio-demographic factors associated with the prevalence of dengue in the eastern part of Sudan in 2011 Infectious Disease epidemiology. BMC Public Health. 2015 Jun 18;15(1).

27. Althouse BM, Hanley KA, Diallo M, Sall AA, Ba Y, Faye O, et al. Impact of climate and mosquito vector abundance on sylvatic arbovirus circulation dynamics in senegal. American Journal of Tropical Medicine and Hygiene. 2015 Jan 1;92(1):88–97.

28. Attaway DF, Jacobsen KH, Falconer A, Manca G, Rosenshein Bennett L, Waters NM. Mosquito habitat and dengue risk potential in Kenya: alternative methods to traditional risk mapping techniques.

29. Mazaba-Liwewe ML, Siziya S, Monze M, Mweene-Ndumba I, Masaninga F, Songolo P, et al. First sero-prevalence of dengue fever specific immunoglobulin G antibodies in Western and North-Western provinces of Zambia: A population based cross sectional study. Virol J. 2014 Jul 30;11(1).

30. Kamgang B, Ngoagouni C, Manirakiza A, Nakouné E, Paupy C, Kazanji M. Temporal Patterns of Abundance of Aedes aegypti and Aedes albopictus (Diptera: Culicidae) and Mitochondrial DNA Analysis of Ae. albopictus in the Central African Republic. PLoS Negl Trop Dis. 2013;7(12).
